# Silico-tuberculosis amidst COVID-19 pandemic: global scenario and Indian perspective

**DOI:** 10.1097/MS9.0000000000001471

**Published:** 2023-11-01

**Authors:** Priyanka Roy, Mainak Bardhan, Shubhajeet Roy, Utkarsh Singh, Timil Suresh, Ayush Anand

**Affiliations:** a Deputy Chief Inspector of Factories/ Deputy Director (Medical) and Certifying Surgeon, Directorate of Factories, Department of Labour, Government of West Bengal, West Bengal; b Miami Cancer Institute, Baptist Health South Florida, FL, USA; c Faculty of Medical Sciences, King George’s Medical University, Lucknow, India; d BP Koirala Institute of Health Sciences, Dharan, Nepal

**Keywords:** COVID-19, occupational health, silicosis, silico-tuberculosis, tuberculosis

## Abstract

Inhalation of crystalline silica-rich dust particles can result in the deadly occupational lung disorder called silicosis. The risk of contracting tuberculosis (TB) and the potential for lung cancer increase due to silicosis. This review article aims to bring to light the state of silicosis and TB scenario in the world and India for evaluating hurdles in the present and future to achieve the elimination road map and assess these conditions in the backdrop of the COVID-19 pandemic. A patient with silicosis has a 2.8–2.9 times higher risk of developing pulmonary TB and 3.7 times that of extrapulmonary TB. Incidences of missed cases when TB was misdiagnosed with silicosis due to indifferent clinical manifestations of the two in the initial stages are not uncommon. The duration of silica exposure and silicosis severity are directly related to the propensity to develop TB. As per a study, an average gap of 7.6 years has been noticed in a South African population for silico-tuberculosis to develop post-silicosis. In a study done on mine workers at Jodhpur, Rajasthan, it was seen that there is no definitive relation between patients with silicosis and the possibility of having COVID-19. There is a significant need to integrate the Silicosis control program with the TB elimination program for the government. A few steps can include assessing the workplaces, periodic monitoring of the workers’ health, active case surveillance, identification of hotspots, and introducing reforms to curb the spread of dust and particulate matter from industrialised areas be taken in this regard.

## Introduction

Highlights

- Coexistence of silicosis and tuberculosis (TB) can be detrimental.
- Silicosis is highly prevalent in mine workers, and the risk of developing TB increases with pre-existing silicosis.
- There is a significant need to integrate the Silicosis control programme with the TB elimination programme.

Silicosis, an often fatal occupational lung disease, is caused by inhaling dust particles laden with crystalline silica (silicon dioxide, a component of sand and quartz) particles^1^. Silica dust can accumulate in the alveolar walls, leading to scarring, which resists its clearance by mucous or coughing, thus hindering gaseous exchange. The symptoms of acute silicosis comprise fever, shortness of breath, and cyanosis, visible more commonly over the ear lobes and the lips. Chronic patients may experience fatigue, extreme shortness of breath, loss of appetite, and chest pain with a risk of respiratory failure. Accelerated silicosis, although rare, is a unique and aggressive form of silicosis that primarily affects those who work with engineered stone. The symptoms tend to progress more quickly than chronic silicosis, often developing within 3–10 years of exposure. Besides lung transplantation, no known treatment exists for halting accelerated silicosis progression^1^. Since silica exposure is commonly occupation-restricted, building and construction workers, miners, sandblasters, ceramics and porcelain manufacturers, marble workers, and demolition experts are particularly susceptible. There are 227 million workers worldwide who are estimated to be most at risk of developing silicosis, mainly migrants. Over 80 nations employ 40.5 million artisanal small-scale miners worldwide^2^. The attributed risk of silicosis in certain occupations can be controlled, but the disease is incurable. Crystalline silica was recently identified as a human carcinogen by the International Agency for Research on Cancer (Group I). The risk of contracting tuberculosis (TB) and the potential for lung cancer increase due to silicosis^1,3^. Hence, the National Institute for Occupational Safety and Health (NIOSH) stated, “*Silica is not just dust, but it is dangerous dust.”*

TB, on the other hand, is an infectious bacterial disease caused by the *Mycobacterium tuberculosis* complex^4^. The endogenous risk factors contributing to the development of progressive symptoms include compromised cell-mediated immunity, senility, and gender, wherein women of 25–34 years are more susceptible, while men face greater risk at older ages^4,5^. HIV positive, genetically predisposed, smokers, intravenous drug abusers, infants, elderly, diabetics, and alcoholics are the ones who face the most risk^4,5^. It is transmitted mainly through aerosols and fomites from an infected source and primarily affects the lung parenchyma, leading to pulmonary manifestations^5^. Alveolar macrophages phagocytise the bacteria in the patient’s lungs before invading the underlying epithelium^4^. As the immune system works to fend off the illness, monocytes from surrounding blood arteries create the first stages of a granuloma in this location, a defining feature of TB^4,5^. The caseous centre tends to liquefy and cavitate as it releases thousands of *M. tuberculosis* bacilli into the airway, despite immunologically being confined^4^. The infected lungs generate a cough containing the highly contagious infectious droplet nuclei. The lymphatic system then transports infected macrophages to the pleura, peritoneum, pericardium, genitourinary tract, meninges, and epiphyses of the long bones, lungs, lymph nodes, skin, and other parts of the body, leading to a composite variety of complications. The primary signs and symptoms of pulmonary tubercular aetiology include fever, night sweats, chronic cough, sputum production, weight loss, fatigue, and hemoptysis^4^. Chest X-ray (CXR) and Sputum Cultures for the acid-fast bacilli staining are of utmost diagnostic importance^4^.

Opportunistic infections continue to be the most significant cause of morbidity and mortality among individuals living with HIV, accounting for around 90% of all cases, with opportunistic malignancies accounting for 7% and other causes accounting for the remaining 3%^6^. Mycobacterium TB is the most common pathogenic organism affecting the HIV population, causing both pulmonary and non-pulmonary TB . The general population has a TB incidence of less than 10%, whereas the HIV community has an annual incidence of more than 10%^7^. This review article aims to bring to light the state of silicosis and TB scenario in the world and India for evaluating hurdles in the present and future to achieve the elimination road map of both diseases and assess these conditions in the backdrop of the COVID-19 pandemic.

## Current disease burden of TB

The WHO and the International Labor Organization started a public awareness and preventive campaign in 1995, intending to eradicate silicosis from the planet by 2030. Although the elimination targets seem virtually impossible to be met, it is essential to note that silicosis has been recognised as an important predisposing factor for TB, next only to HIV infection^8^. In 2019, there were over 12 900 deaths reported from silicosis worldwide, and 655 700 silicosis-related DALYs were recorded. Middle Socio-Demographic Index countries recorded 5500 mortalities with a crude death rate of 0.23 in 2019^9^. According to WHO, an estimated 10.6 million people contracted TB, while 1.6 million persons worldwide died in 2021 (including 187 000 HIV-positive patients)^10^. Following COVID-19, TB is the second most infectious fatal aetiology globally and the 13th largest overall cause of mortality. The areas of Southeast Asia (44%), Africa (25%), and the Western Pacific (18%) reported the highest numbers of TB cases. Eight countries made up the majority of the global total: India (26%), Indonesia (8.5%), China (8.4%), the Philippines (6%), Pakistan (5.7%), Nigeria (4.4%), Bangladesh (3.6%), and South Africa (3.6%). While there has been some progress, it has been prolonged, and it is anticipated that the globe will not be able to meet the End TB Strategy’s target of completely eradicating TB as a threat to public health by 2035. For instance, the planned incidence reduction during 2015–2020 was 20% but could be limited to only 9%. Simultaneously, the projected mortality rate reduction for the same period was 35%, where only a 14% change could be managed^11^.

## Coexistence of TB and silicosis

### Prevalence

It is challenging to estimate the global prevalence of silico- TB since there is inadequate surveillance and access to medical treatment, and how many people in resource-poor countries contract the disease is unclear^12^. Due to insufficient safety laws, a lack of preventative instruments, and lower levels of knowledge, low-income countries have a higher incidence of silicosis and silico- TB^13^. A survey in the Indian state of Rajasthan on a group of 174 mine workers, where the mean age was 39.13±11.09 years and among which three-fourths were mining for more than 10 years, reported a history of TB in 30% of them. It was found that 10.0% of miners had contracted TB, 7.4% had developed silico- TB, 37.3% showed features of silicosis, and 4.3% had other respiratory illnesses such as emphysema and pleural effusion^14^. A similar study conducted in the sandstone mines of Rajasthan evaluated 529 CXRs of mine workers. 275 (52%) CXRs presented radiological evidence of silicosis. 61 (12.4%) of the silicosis patients also developed tubercular changes, known as silico- TB^15^. A recent meta-analysis showed that silicosis in parts of Zimbabwe and Egypt had a threefold higher chance of developing TB^15^. Due to the increment in the stone benchtop industry and handling of artificial stone, there is an increased exposure of silica to mine workers, which also increases the risk of other pulmonary diseases, especially TB. A PubMed Central search was conducted using the keywords “silicotuberculosis” and “prevalence” and the time period of the last 20 years, which yielded 15 studies, out of which only seven were found to be relevant in terms of exemplifying the prevalence of silico-TB at various geographical locations around the world. (Table 1)^12,15–20^.

**Table 1:** Burden of silico-tuberculosis at various geographical locations.

S.No.	Study title (authors)	Year of publication	Study site	Participants	Corresponding occupation / line of work	Prevalence of silico-tuberculosis
1	Tuberculosis and Silicosis Burden in Artisanal and Small-Scale Gold Miners in a Large Occupational Health Outreach Programme in Zimbabwe (Moyo *et al.* ^14^)	2021	Zvishavane, Gweru, Insiza and Gwanda districts in the Midlands and Matabeleland South provinces, Zimbabwe	514	Artisanal and Small Scale Miners	2.2%
2	The Triple Burden of Tuberculosis, Human Immunodeficiency Virus and Silicosis among Artisanal and Small-Scale Miners in Zimbabwe (Moyo *et al.* ^15^)	2022	Zvishavane, Insiza, Gwanda, Gweru, Chirumhanzu, Kwekwe and Shurugwi districts in the Midlands and Matabeleland South provinces, Zimbabwe	3821	Artisanal and Small Scale Miners	2.5%
3	Silicosis, progressive massive fibrosis and silico-tuberculosis among workers with occupational exposure to silica dusts in sandstone mines of Rajasthan state: An urgent need for initiating national silicosis control programme in India (Nandi *et al.* ^16^)	2021	Karauli and Dholpur districts, Rajasthan, India	529	Sandstone Mine Workers	12.4%
4	Silico-tuberculosis and associated risk factors in central province of Iran (Farazi *et al.* ^17^)	2015	Markazi province, Iran	3121	Mining, Mineral Processing, Stone Cutting, Pottery, Glass Manufacturing, Masonry, Rock Drilling, Sand and Gravel Work	0.9%
5	Respiratory Health of Female Stone Grinders with Free Silica Dust Exposure in Gujarat, India (Tiwari *et al.* ^18^)	2013	Chhotaudepur village of the Godhra district of Gujarat, India	85	Quartz Mill Stone-Grinding	11.6%
6	Cost-benefit analysis of installing dust control devices in the agate industry, Khambhat (Gujarat) (Bhagia *et al.* ^19^)	2008	Khambat, Gujarat, India	1126	Agate Grinders	20.0%
7	Comparison of respiratory morbidity between present and ex-workers of quartz crushing units: Healthy workers’ effect (Tiwari *et al.* ^20^)	2010	Godhara region of Gujarat, India	316	Quartz Stone Grinders	24.6%AA

### Risk factors

Exposure to silica is also linked to an increased risk of developing silicosis as well as pulmonary and extrapulmonary TB^21^. It can last years after exposure, increases with disease severity, and is more common in acute and rapid silicosis patients^22,23^. According to reports, silicosis patients have a 2.8–2.9 times higher chance of acquiring pulmonary TB than the general population and a 3.7 times greater risk of developing extrapulmonary TB^24^. In a study involving 2255 South African gold miners who were followed up for 24–27 years, the length of exposure and the severity of silicosis (where present) were associated with the chance of getting pulmonary TB. Moreover, the study found a mean gap of 7.6 years between the cessation of silica dust exposure and the diagnosis of pulmonary TB^22^. HIV infection, another potent risk factor for TB, causes increased reactivation of the latent *Mycobacterium*, thus increasing the incidence and progression of the disease. A lack of early diagnosis and treatment regimens may contribute to a rise in the rate of TB infection. In contrast, prior TB therapy may be linked to more than half of individuals acquiring silico-TB, increasing with age (>40 y), which may further increase the infection rate^25^.

### Presentations

**Figure 1:**
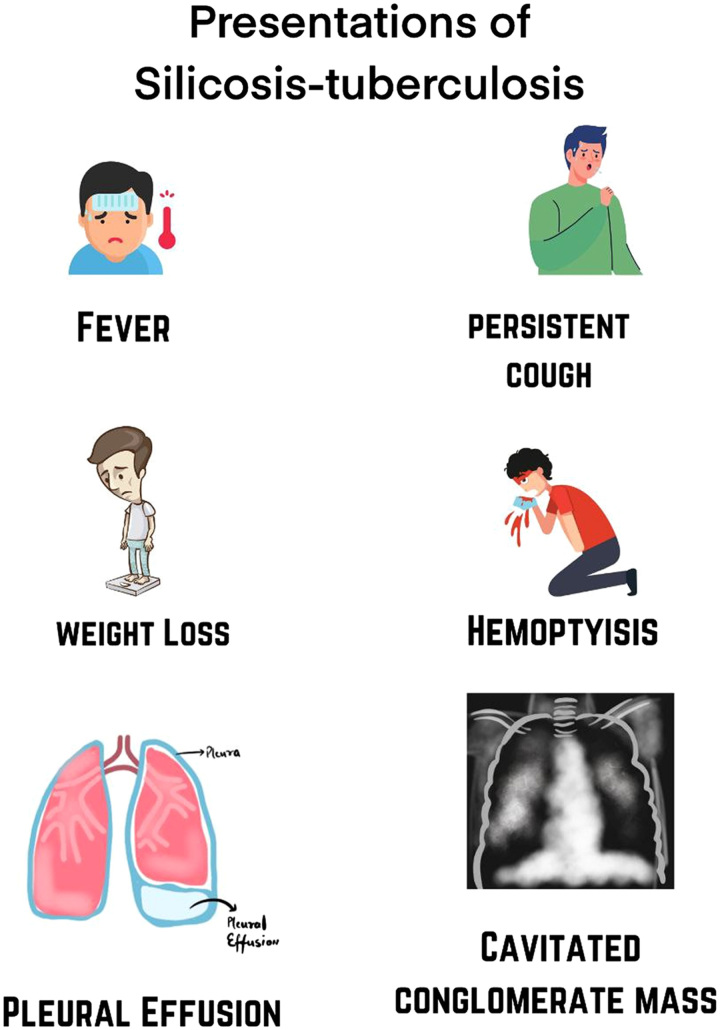
Clinical presentation of silico-tuberculosis.

Figure 1 shows the various presentations of silico-TB. Regarding silico-TB, the prognosis is primarily influenced by the tuberculous component. In the early stages, the presence of typical symptoms of pulmonary TB, such as fever, persistent cough, weight loss, or hemoptysis, does not provide substantial evidence of any coexisting silicosis in the patient since these clinical features are not distinguishable from pre-existing silicosis. Additionally, new opacities appear rapidly on radiological imaging, and pleural effusion or excavations may be observed. The presence of a cavitated conglomerate mass strongly suggests silicosis associated with TB. Although detecting active disease clinically in patients with silicosis is difficult, some patients may exhibit symptoms of constrictive pericarditis in later stages^26–28^. Primarily, after exposure over 10–30 years, the patients slowly and insidiously develop progressive nodular, fibrosing pneumoconiosis, which is characterised by the accumulation of abundant lipo-proteinaceous material inside the alveoli, which can be very much similar to the findings of alveolar proteinosis. Dose and race are believed to have a role to play in developing the symptoms, with African Americans at higher risks than Caucasians. Several studies have shown that with prolonged exposure to silica, there are high chances for the development of complications like broncholithiasis, pneumothorax, and aspergillosis^1,29,30^. In rare cases, broncho-oesophageal fistulas are seen in workers with TB and pre-existing silicosis^31^. Silicosis patients are prone to TB because the crystalline silica inhibits the pulmonary macrophages from destroying the phagocytosed bacilli^32^. Respiratory droplets mainly transmit SARS-CoV-2, while touch, aerosols, and fomites are possible transmission routes. The median incubation time for COVID-19 infection is expected to be 5.1 days (95% CI, 4.5–5.8 days), and 97.5% of those infected experience symptoms within 11 days (95% CI, 8.2–15.6 days) following exposure^33,34^. Fever and chills, cough, dyspnoea, exhaustion, myalgia, headache, loss of taste and smell sense, sore throat, rhinorrhea, nausea, vomiting, and diarrhoea are among the symptoms^35^. TB can also present as an opportunistic infection in patients with HIV. In a study by Mohamud and colleagues, people living with HIV/AIDS in Mogadishu, Somalia, are at risk of Opportunistic Infections, particularly those who live with domestic animals, have a chronic co-morbid condition, consume untreated water, and have poor ART compliance. Furthermore, pulmonary TB was the most frequent opportunistic infection in this cohort^36^.

The prevalence of Opportunistic Infections varies according to the patient’s profile and risk factors, which include education level, gender, resident region, occupation, hygiene, and nutritional status. Other risk factors contributing to the prevalence of Opportunistic Infections include a weakened immune system due to advanced age, certain medications such as immunosuppressants, and exposure to environmental pollutants. Additionally, individuals with a history of substance abuse or those living in crowded and unsanitary conditions are more susceptible to these infections. Geographical differences exist in the severity and most prevalent types of opportunistic infections^37–40^. Oesophageal candidiasis, TB, herpes simplex virus, and cryptosporidiosis all decreased in the United States, but pneumonia climbed^37^. In Spain, oesophageal candidiasis and TB reduced dramatically, but the *Mycobacterium avium* complex increased^41^. Low-education persons in Brazil have a high prevalence of TB and toxoplasmosis^7^.

### Diagnosis

CXRs and sputum samples are the mainstay of the initial evaluation of pulmonary TB and silicosis^1,4,5,42^. Sputum testing is less effective than routine radiography screening for early detection. The most significant sign of silico-TB is cavitation, which may result from ischaemic abnormalities in the fibrotic mass of silicosis^42^. CXRs of Silicosis patients show fine nodularity, mainly in the upper part of the lungs. In addition, a computerised tomography scan can be of significant diagnostic value^43^. During the initial phase of the disease, pulmonary function is either unaffected or moderately affected. Only when there is progressive massive fibrosis does shortness of breath develop. The disease remains in progression, even after the exposure to silica is curtailed. So, due to suboptimal pulmonary function, there are severe limitations to a person’s activity, although death might be a very late outcome^42^. Hence, spirometry can be performed to assess lung function impairment. Certain biomarkers are the more advanced diagnostic options available. A study conducted by the Indian Council of Medical Research revealed that Club Cell protein 16 (CC16), which is a potential anti-inflammatory protein secreted by non-ciliated bronchoalveolar epithelium, is found to be reduced in case of long-term exposure to silica and hence can be used as an early detection tool. Certain other biomarkers, like Neopterin, serum selenium, haem oxygenase, etc., can also be used for detection^44^. Cases of interstitial lung diseases (ILDs), or ILDs with TB, are frequently misdiagnosed and treated with antitubercular therapy alone. In a study from Karachi, Pakistan, it was seen that 3 out of 3 (100%) (two had sputum-positive TB in the past, i.e. they had silico-TB) silicosis patients were initially treated as TB. The authors further say that a great degree of clinical suspicion is needed to diagnose ILDs. Cough and dyspnoea, with which the patients most commonly present, are overlooked in most cases and are erroneously thought to be due to a history of smoking, increasing age, or some other co-infections, the most common of which is TB. The reason behind this is the less aware physicians. Due to such instances, patients are subjected to unnecessary ATT treatments, which carry a spectrum of side effects, and the disease remains undiagnosed. All these lead to increased mortality rates in such groups of patients^45^. Molecular identification of SARS-COV-2 by polymerase chain reaction of nasopharyngeal specimens, deep nasal swabs, throat swabs, or lower respiratory samples is the best method for precise diagnosis^46^.

### Management

To reduce the risk of recurrence, standard anti-TB medications with directly observed treatment are advised to be used for at least eight months. The chemotherapy used in TB patients has proved equally effective in silico-TB patients^21^. The introduction of CC16 protein has highly improved the management of silico-TB as a potential biomarker for silicosis, which helps in deciding the treatment regimen for the patients. Patients with silicosis must be monitored for developing or redeveloping TB. The emphasis should also be on preventing the incidence of silico-TB and uplifting the quality of the workplace for workers working in silica industries. These measures include decreasing the exposure to silica dust, ensuring completion of anti-tuberculosis treatment, reducing oscillating migration of workers, compensating workers properly, training and educating occupational health to the working community, alleviating the quality of life of labourers, intensifying medical surveillance system and TB screening during scheduled health check-ups, which should be made mandatory to be conducted by the mine or factory owners or the government, as the case might be, and proper policy framing to reduce inhalation of dust by workers or employees, to bring down the incidence of silico-TB^47^. Patients with mild disease should self‐isolate, and can often be managed in the community by home-based care. They must also be able to monitor their health, know which signs call for a doctor’s attention, and know how to voice any concerns. In general, hospitalisation is necessary for patients with moderate to severe sickness. This includes people with extensive pulmonary infiltrates visible on chest imaging, tachypnoea at rest (respiratory rate >22 breaths/min), who are dyspnoea on light exertion, hypoxaemia, and acutely altered mental status. The requirement for immediate admission and consideration of intensive care, if necessary for a particular patient, are dictated by severe sickness, which is indicated by, among other aspects, a respiratory rate greater than 30 breaths/min, SpO2 of 92% on room air, or prolonged hypotension. Treatment includes antivirals like Nirmatrelvir with Ritonavir, Remdesivir, and Molnupiravir. Immunocompromised patients or those on immunosuppressants may also benefit from convalescent plasma therapy.

In a study conducted by the Hong Kong Chest Service/Tuberculosis Research Centre and the Madras/British Medical Research Council, patients with silico-TB were randomized to receive either 6 months or 8 months of three-times-weekly therapy with isoniazid, rifampicin, pyrazinamide, and streptomycin in a controlled clinical trial in Hong Kong. Patients with prior anti-TB therapy history also received ethambutol for the first three months. In two months, 80% of patients converted to a negative sputum culture, 22% of patients treated for 6 months relapsed 3 years later compared to 7% treated for eight months, and 22% of patients experienced significant medication side effects^48^. In a subsequent South African study, gold miners with pulmonary TB were given isoniazid, rifampicin, pyrazinamide, and streptomycin on weekdays for five months. Chest X-rays were evaluated for silicosis at the time of diagnosis, and all participants were followed up for at least 5 years after treatment was completed. The probability of TB recurrence was 1.55 times greater in individuals with silicosis than in patients without silicosis^49^.

Lung transplantation is the ultimate and lone treatment option for end-stage silicosis. But, the literature points towards the fact that patients with silicosis who underwent lung transplantation (4.9%) had a nonstatistically significant survival advantage (hazard ratio: 0.6), contrasted to those undergoing the same for idiopathic pulmonary fibrosis^50^.

#### Impact of COVID-19 on silicosis and silico-TB

Silicosis and silico-TB are lung diseases caused by long-term exposure to crystalline silica dust in the workplace. The COVID-19 pandemic has raised concerns about the impact of the virus on these diseases, which already pose significant health risks to workers in high-risk occupations.

### Reduced access to healthcare

The pandemic has strained healthcare systems worldwide, reducing access to healthcare services. Workers suffering from silicosis and silico-TB may find it challenging to access healthcare services, including diagnosis and treatment^51^. This could lead to delayed diagnosis and progression of these diseases, resulting in poorer outcomes. There was a substantial drop in patients from primary and workplace-based occupational healthcare during COVID-19^52^. India, a country with a high prevalence of silicosis and TB, found that the pandemic has reduced access to healthcare services for these patients, leading to delays in diagnosis and treatment. The shortage of healthcare workers led to inadequate healthcare for COVID-19-affected patients^53^. According to the International Labour Organization, lockdown measures have touched 2.7 billion individuals, or 81% of the world’s employment, with 61% of employees belonging to informal sectors in poor and middle-income countries. Inadequate healthcare access with no economic protection made those workers more vulnerable and marginalised^54^.

### Increased risk of exposure

The COVID-19 pandemic raised the demand for personal protective equipments (PPEs), such as respirators. In some places, this resulted in a lack of PPE, increasing the danger of workers being exposed to silica dust^55^. Workers may also be reluctant to wear PPEs due to discomfort and the increased risk of contracting COVID-19 when wearing a respirator for long periods. Also, following social distancing may not be a practical solution for mine workers.

### Changes in working patterns

The pandemic has also led to changes in working patterns, such as remote working, which may reduce some workers’ exposure to silica dust. However, essential workers beyond healthcare were forced to work at factories and other enterprises, often without providing PPEs^54^. Migrant workers had to return to their homes during the lockdown, mainly in rural areas, spreading the viral infection from urban ‘hotspots’ to the villages. However, the existing healthcare institutions did not have enough infrastructure for testing, tracking, and preventing the disease^56^.

### Compromised immune systems

People with silicosis and silico-TB may have compromised immune systems, increasing their susceptibility to COVID-19^32,57,58^. A study from South Africa found that the SARS-COV-2 infection rate among mine workers at high risk for silicosis and silico-TB exceeded the normal population. Existing ILDs have been associated with severe COVID-19 complications^59^. A multicentric study in the USA showed that individuals with ILD are at increased risk of morbidity and mortality from COVID-19 infection^60^. A study from India showed that subjects from silicosis-prone areas were not at a higher risk of COVID-19, possibly due to pre-existing inflammation^61^. Further studies are needed to assess the long-term impact of the pandemic on these diseases. COVID-19 may potentially exacerbate the risks and prevalence of silicosis and silico-TB by reducing healthcare access, increasing exposure risk, and compromising immune systems. It is crucial to ensure that workers in high-risk occupations have access to adequate healthcare and PPEs to minimise the risk of exposure to silica dust and to prevent the spread of COVID-19.

## Conclusion

This paper aimed to shed light on the coexistence of silicosis and TB in workers exposed to silica dust. Silicosis is a fatal occupational disease that is highly prevalent in mine workers, and the risk of developing TB increases with pre-existing silicosis. There is no known definite treatment for accelerating silicosis except a lung transplant. TB is becoming a burden among resource-poor countries. The silicosis affected labourers, especially mine workers, who are at increased risk of contracting TB. The presentation of silicosis TB includes persistent cough, fever, weight loss, and hemoptysis. With due course of time, the patient can develop fibrosing pneumoconiosis. Broncholithiasis, pneumothorax, and aspergillosis,. The mainstay of the initial evaluation of silicosis TB chest X-Rays and sputum samples. As far as the impact of COVID-19 is concerned, the mine workers being financially weakened, had limited access to healthcare, their exposure to silica dust was increased due to lack of PPE, and some were unwilling to wear it. COVID-19 had a significant impact on their immune systems. The mine workers are working in an already hazardous environment, so their immune systems are highly prone to get compromised hence, there is a significant need to integrate the Silicosis control program with the TB elimination program for the government to improve the health status of mine workers and achieve the goal of TB elimination from the country.

### Future recommendations

The honourable National Human Rights Commission of India has directed and recommended the Governments of the states and union territories to provide complete information regarding the magnitude of the silicosis problem and action taken to prevent and mitigate that disease. Ministry of Health and Family Welfare has already identified the comorbidity of TB-HIV, TB and diabetes, TB and nutrition, TB and tobacco^62,63^. Still, TB and silicosis’s comorbidity is not yet determined^62,63^. As silicosis prevention is identified as a national challenge, this is high time to work on silico-TB. Also, a Silicosis prevention, treatment and rehabilitation policy is needed. Rajasthan came up with this policy first in India, followed by the state of Haryana and West Bengal. Silicosis is an age-old occupational disease that remains challenging when it coexists with TB. The success of prevention and mitigation of both issues needs stakeholders’ active cooperation. A systematic mechanism needs to be placed under the Department of Labour, Department of Health and Family Welfare, and other agencies dealing with TB to address the problem for a healthy and productive workforce and to alleviate human suffering.

**Figure 2:**
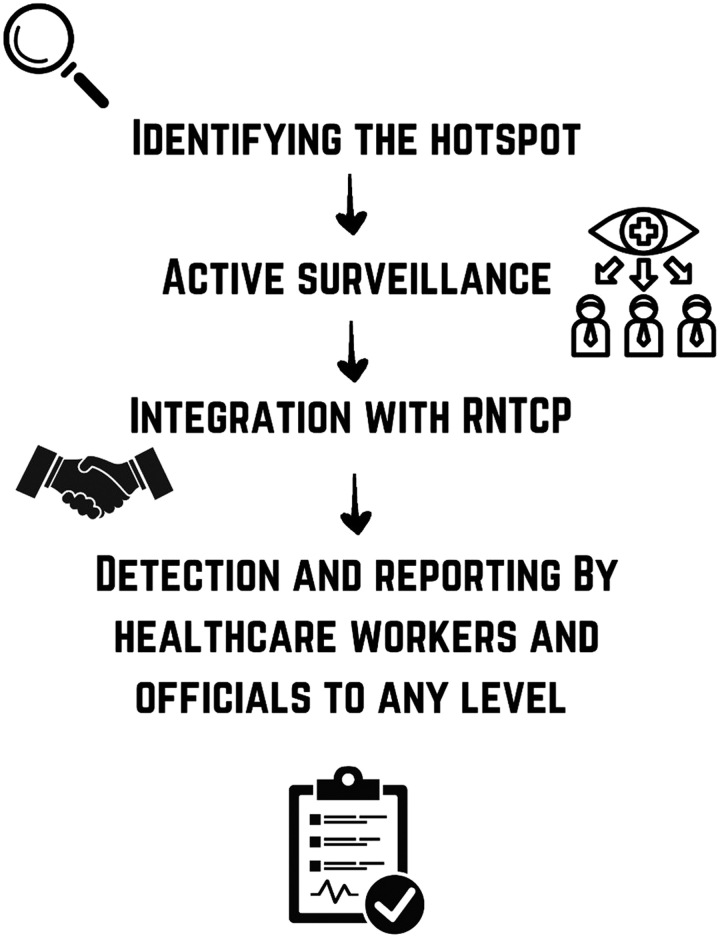
Silicotuberculosis detection and reporting steps within the prevalent similar system for tuberculosis.

The risk assessment of the workplaces where silica dust is abundant should be done regularly, and the local bodies should monitor and grade such reports. The occupational history taking should be mandatory before screening for TB to minimise the risk of misdiagnosis. In this regard, private sector firms and the government must come together and work towards framing a set pattern of rules. Tracking workers’ health periodically at silica dust-prone places should be prioritised. Efforts should be made to incorporate and integrate such actions with the ongoing Revised National Tuberculosis Control Program. The strict vigilance of statutory body should be ensured because silicosis is notifiable and compensable occupational disease under the Factories Act 1948, the Mines Act 1952, the Building and Other Construction Act 1996, Employees’ State Insurance Act 1948. Active surveillance to detect cases of silico-TB should be taken up as one of the urgent goals in hotspot areas of Silica dust production to ensure active case detection, that too in the early stages of the disease pathogenesis. It is imperative to identify such hotspots at all levels, beginning right from the ward level and escalating to the block level, followed by the city level, division level, district level, state level and ultimately, at a pan India level. Workforce and officials who are specially trained and deputed for silico-TB detection and reporting should be employed at all levels within the prevalent similar system for TB (Fig. 2). It should be made imperative for the government and local councils of a state or a city to introduce reforms to curb the spread of dust and particulate matter from industrialised areas. The Delhi Government took one such initiative to keep in check the steadily decrementing Air Quality Index of the Indian Capital. The ruling made construction sites over 20 000 sq. feet mandatory to install at least three air quality monitoring systems to control dust pollution and limit workers’ time in potentially exposure-laden environments. These systems will detect PM2.5 (finer particles—more invasive) and PM10 (coarse particles—less invasive) particles being emitted and will report the data to the Delhi Pollution Control Committee for analysis and effect^51^. In India, inadequate primary healthcare facilities in rural parts of several states, unregulated private healthcare, a lack of political will, and administrative loopholes are significant impediments to TB management. To prevent silicosis, we need a robust database of workplace and health profile of silica exposed workers to identify the magnitude of the problem and vulnerable working group.

## Ethical approval

Ethics approval was not required for this review.

## Consent

Informed consent was not required for this review.

## Source of funding

The authors did not receive funding for this work.

## Author contribution

P.R.: conceptualization, project administration, supervision, writing—original draft, writing—review and editing. M.B.: conceptualization, project administration, supervision, writing—original draft, writing—review and editing. S.R.: Conceptualization, writing—original draft, writing—review and editing. U.S.: Conceptualization, writing—original draft, writing—review and editing. T.S.: Conceptualization, writing—original draft, writing—review and editing. A.A.: Project administration, supervision, writing—original draft, writing—review and editing.

## Conflicts of interest disclosure

None.

## Research registration unique identifying number (UIN)

None.

## Guarantor

Priyanka Roy is the Guarantor.

## Data availability statement

Not applicable.

## Provenance and peer review

Not commissioned, externally peer-reviewed.
